# New Inventions

**Published:** 1912-07

**Authors:** 


### New Inventions

Wilhelm Hiemenz and Walter Kropp, Elberfeld, Ger. Assignors to Farbenfabriken Vorm, Friedr Bayer & Co., Elberfeld, Ger. 1,026,914. Pharmaceutical Product.

L. H. Reuter, Tompkinsville, N. Y. 1,027,000. Composition for Disinfecting and other purposes.

B. B. Goldsmith, New York, N. Y. 1,027,131. Indurated Albuminoid Compound.

B. B. Goldsmith, New York, N. Y. 1,027,122. Indurated Casein Compound.

Louis Block, New York, N. Y. Assignor to Leah E. Block, New York, N. Y. 1,027,174. Earpiece-Disinfector.

J. B. Sapp, Cleveland, Ohio. 1,027,216. Obtunder.

Geo. Merling and Hugo Kohler, Elberfeld, Ger. Assignors to Farbenfabriken Vorm, Friedr, Bayer & Co., Elberfeld, Ger. 1,026,691, 1,026,692. Process of Producing Isoprene.

J. M. Brandenburg, Elgin, Ill. 1,026,786. Corn Plaster.

W. H. Redington, Evanston, Ill. 1,026,830. Stopper.

A. J. Kier, Copenhagen, Den. 1,026,956. Apparatus for filling Tubes.

Edw. Dobartz & W. H. Sigley, Slyvan Grove, Kansas 1,027, 378. Funnel.

Robt. Liddell, Tecumseh, Nebr. 1,029,892. Hydrocarbon Burner.

H. T. Smith, Willis, Kans. Assignor to B. L. Cornelius, Hutchinson, Kansas, & A. V. Smith, Willis, Kansas,. 1,028,973. Oil Burner.

A. R. Swaine, Des Moines, Iowa. 1,028,976. Hydrocarbon Burner.

A. H. Schilke, Wilmerding, Pa. 1,029,031. Combination Burner and Blowpipe.

J. B. Cowper, Bayonne, N. J. 1,029,048. Oil Burner.

Alexander J. Rickie, Cossipur, Calcutta, India. 1,029,084. Gas Producer or Generator.

Emil Lamphecht, Phila., Pa. 1,029,135. Gas Lighter.

L. A. Voell, Fund du Lac, Wis. 1,029,158. Igniting Device.

H. M. Doggett, Oakland, Cay. 1,029, 174. Oil Burner.

J. W. Aylsworth, East Orange, N. J. Assignor to F. L. Dyer, Montclair, N. J. 1,029,254. Non Inflammable Lubricating Oil.

A. I. Blanchard, Islington, London, England. 1,029,264. Vapor Burner.

C. W. Miles, Anderson township, Hamilton County, Ohio. Assignor to S. S. Miles, Greenboro, N. C. 1,029.309. Air and Gas Compressor.

R. L. Spaulding, Helena, Mont. 1,028,297. Sterilizer.

Arthur Zitcher and E. J. Rath, Offenbach-on-the-Main, Germany. Assignors to Corporation of Chemische, Fabrik Griesheim Elektron, Frankfort-on-the-Main, Germany. 1,028,521. Anthracene Derivatives and Process of Making Same.

E. I. McKesson, Toledo, Ohio. 1,028,582. Anesthetic-Administering Apparatus.

E. I. McKesson, Toledo, Ohio. 1,028,583. Rebreathing-Bag for Anesthetizing Apparatus.

G. F. Bridley, San Francisco, Cal. Assignor to The Roessler & Hasslacher Chemical Co., New York, N. Y. 1,028,857. Apparatus for Preparing Ozone.

Henry Howard, Boston, Mass. 1,028,880. Process for Mak-Sulfuric Anhydrid.

John Leach, Blackburn, Eng. 1,028,890. Medical Inhaler.

Joseph Rosenthal, Munich, Ger. 1,028,969. X-Ray Tube.

Chas. Endorf, Jr., Chicago, Ills. Assignor to Commercial Electric Motor Mfg. Co., Chicago, Ills. 1,028,999. Massage Apparatus.

Josef Stoger, Munich, Ger. 1,029,034. Obstetrical Device.

G. F. Jaubert, Paris, France. 1,029,064. Process for the Preparation of Hydrogen by Autocombustion.

G. L. Clarke, Brooklyn, N. Y. Assignor to The Eden Co. 1,029,105. Prefume Preparation.

Carl Bosch and Hans Keller, Ludwigshafen-on-the-Rhine, Ger. Assignor to Badische Anilin & Soda Fabric, Ludwigshafen-on-the-Rhile, Germany. 1,029,528. Process of Absorbing Oxids of Nitrogen.

Paul Hoering, Berlin, Ger. 1,027,844. Process of Making a Soluble Compound of Iron-Glycero-Phosphate Combined with Milk or Milk-Albumin.

Carl Von Linde, Munich, Ger. 1,027,862, 1,027,863.

Arthur Quenzer New York N. Y. 1,027,897. Ankle-Supporter.

C. C. Beamer, Steubenville, O. 1,027,976. Medicine-Spoon.

Albrecht Schmidt, Hochst-on-the-Main, Ger. Assignor to Farbwerke Vorm, Meister Lusius & Bruning, Hochst-on-the-Main, Ger. 1,027,441. Isolated Alkali Salts of Indoxyl and Process of Making Same.

W. G. Lindsay, New York, N. Y. Assignor to The Celluloid Co., New York, N. Y. 1,027,486, and various other numbers. Solvents for Acetyl Cellulose.

W. G. Lindsay, New York, N. Y. Assignor to the Celluloid Co., New York, N. Y. 1,027,617, 1,027,,618. Solvent for Nitrocellulose.

G. D. Hazen, Brockton, Mass. Assignor to Hazen-Brown Co., Brockton, Mass. 1,027,728. Collapsible Tube.

Georg Zueler, Berlin, Ger. Assignor to Chemische Fabrik Auf Actien (Vorm E. Schering), Berlin, Ger. 1,027,790. Pancreas Preparation Suitable for the Treatment of Diabetes.

R. H. Davis, London, Eng. Assignor to Siebe Gorman and Co., Ltd., London, Eng. 1,027,823. Apparatus for Testing Air.

